# Welchen Anteil hat die Infektiologie am Fachgebiet Urologie? Ein Pilotprojekt, zur Hervorhebung der Antibiotic-Stewardship-Bedeutung

**DOI:** 10.1007/s00120-022-01898-1

**Published:** 2022-07-13

**Authors:** Laila Schneidewind, Fabian P. Stangl, Desiree L. Dräger, Florian M. E. Wagenlehner, Oliver W. Hakenberg, Jennifer Kranz

**Affiliations:** 1 Urologische Klinik und Poliklinik, Universitätsmedizin Rostock, Schillingallee 35, 18055 Rostock, Deutschland; 2 Universitätsklinik für Urologie, Inselspital Bern, Bern, Schweiz; 3 Klinik für Urologie, Kinderurologie und Andrologie, Justus-Liebig-Universität-Gießen, Gießen, Deutschland; 4 Klinik für Urologie und Kinderurologie, Uniklinik RWTH Aachen, Aachen, Deutschland; 5 Universitätsklinik und Poliklinik für Urologie, Universitätsklinikum Halle (Saale), Halle (Saale), Deutschland

**Keywords:** Infektiologie, Harnwegsinfektionen, Sepsis, Antibiotic Stewardship, Resistenzentwicklung, Infections in urology, Urinary tract infections, Sepsis, Antibiotic stewardship, Antimicrobial resistance

## Abstract

**Hintergrund:**

Harnwegsinfektionen gehören weltweit zu den häufigsten bakteriellen Infektionskrankheiten, sowohl im ambulanten als auch stationären Setting.

**Fragestellung:**

Mit diesem Pilotprojekt soll primär die Frage beantwortet werden, welchen relativen Anteil der Antibiotikaeinsatz zur Therapie bakterieller Infektionen in einer universitären Urologie hat, um Antibiotic-Stewardship(ABS)-Programme besser implementieren zu können.

**Material und Methoden:**

Es handelt sich um ein epidemiologisches Pilotprojekt. Über einen Zeitraum von insgesamt drei Monaten wurde systematisch erhoben, wie viele Patientinnen und Patienten relativ im Verhältnis zur Gesamtanzahl der Patientinnen und Patienten eine Antibiotikagabe erhielten.

**Ergebnisse:**

Bei rund 40 % der urologischen Patientinnen und Patienten einer universitären Einrichtung wurde eine antimikrobielle Therapie bei bakterieller Harnwegsinfektion bzw. eine perioperative Antibiotikaprophylaxe zur Vermeidung bakterieller Komplikationen im Operationssaal eingesetzt. Insgesamt ist der Antibiotikaeinsatz im stationären Bereich am höchsten.

**Diskussion:**

Infektiologische Aspekte haben am Fachgebiet Urologie einen signifikanten Anteil. Dieses Wissen ist essentiell um ABS-Programme erfolgreich zu implementieren und der Resistenzentwicklung entgegen zu wirken. Detailliierte Folgeuntersuchungen sind notwendig, um die Antibiotikaverordnungspraxis in der Urologie genau zu verstehen und daraus gezielte ABS-Interventionen entwickeln zu können.

## Abstract (en)

**Background:**

Urinary tract infections account for one of the most bacterial infectious diseases worldwide.

**Objectives:**

The primary aim of this pilot project was to identify the relative percentage of antibiotic use in comparison to all patients in a university medical center for the better establishment of antibiotic stewardship (ABS) programs.

**Material and methods:**

This is an epidemiological pilot project. In the time period of three months it was evaluated which relative percentage of the patients was treated with antibiotics for bacterial urinary tract infection in comparison to all patients.

**Results:**

In summary, about 40% of all urological patients received an antibiotic treatment against urinary tract infections or as perioperative prophylaxis against bacterial infection in the operating room (OR). The antibiotic use at the urological ward is highest in comparison to ambulance or OR.

**Conclusion:**

Infectious diseases, especially bacterial infections, account for a significant part of urology. This knowledge is essential to establish ABS programs and to tackle the progression of antibiotic resistance. Detailed studies are necessary to understand antibiotic prescription practice in urology to develop targeted ABS interventions.

## Hintergrund und Fragestellung

Harnwegsinfektionen gehören zu den häufigsten bakteriellen Infektionen in der Gesellschaft und im Gesundheitssystem, jährlich sind weltweit etwa 150 Mio. Menschen betroffen [1–3]. Die Situation stellt sich in Deutschland ebenso dar, auch hier gehören Harnwegsinfekte sowohl im ambulanten als auch im stationären Bereich und bei den gesundheitssystemassoziierten Infektionen zu den häufigsten bakteriellen Infektionskrankheiten [4, 5].

Zusätzlich besteht weiterhin das Problem der zunehmenden Resistenzraten gegenüber Antibiotika, insbesondere im gramnegativen Bereich. Ein besonders dramatisches Bild zeichnet der World Health Organization(WHO)-Bericht „Antimicrobial Resistance Global Report on Surveillance 2014“: In Europa werden durch resistente Bakterien ca. 25.000 Tote und etwa 1,5 Mrd. € Mehrkosten für die Gesundheitssysteme pro Jahr erwartet [6, 7]. Dieser Bericht ist durchaus weiterhin als aktuell zu verstehen.

Urologen sind dabei ein wesentlicher Faktor für das Problem der weiter steigenden Resistenzraten: Laut den GERMAP(Antibiotikaverbrauch und die Verbreitung von Antibiotikaresistenzen in der Human- und Veterinärmedizin in Deutschland der Paul-Ehrlich-Gesellschaft für Infektionstherapie e. V.)-Berichten rangieren Urologen immer wieder im oberen Bereich des Antibiotikaverordnungsvolumens (pro Tagesdosis) als Fachgruppe in Deutschland [6, 8].

Aus diesen Gründen stellt sich konsequenterweise die Frage, welchen Anteil infektiologische Aspekte am Fachgebiet der Urologie überhaupt haben oder anders ausgedrückt: Wie viel Infektiologie steckt in der Urologie? Systematische Untersuchungen zu dieser Fragestellung gibt es unseres Wissens bisher nicht. Allerdings ist dieses Wissen essentiell, um Kollegen für Antibiotic Stewardship (ABS) zu sensibilisieren und bestehende ABS-Programme weiterzuentwickeln sowie zu verbessern, um der antimikrobiellen Resistenzentwicklung entgegen zu wirken.

Die ABS-Programme haben das Ziel, die Qualität der Verordnung von Antiinfektiva bzgl. Auswahl der Substanzen, Dosierung, Applikation und Anwendungsdauer kontinuierlich zu verbessern, um beste klinische Behandlungsergebnisse unter Beachtung einer Minimierung von Toxizität für den Patienten, Resistenzentwicklung und Kosten zu erreichen. Die ABS-Kernstrategien sind dabei z. B. die Erstellung und Aktualisierung lokaler Therapieleitlinien und Behandlungspfade, Erstellung einer Antiinfektivahausliste unter Berücksichtigung nationaler und internationaler Leitlinien sowie lokaler/regionaler Erreger- und Resistenzlage, gezielte Fortbildung, Schulung und Information, proaktive Analysen von Antiinfektivaverordnungen und -visiten vor Ort sowie die Integration der ABS-Programme in die einrichtungsspezifische Qualitätssicherung [6, 9, 10].

Daher soll in diesem Pilotprojekt primär die Frage beantwortet werden, welchen relativen Anteil der Antibiotikaeinsatz zur Therapie und Prävention bakterieller Infektionen in einer universitären Urologie im Zeitraum von drei Monaten hat, um abschätzen zu können, welchen Anteil infektiologische Aspekte am Fachgebiet Urologie haben.

## Studiendesign

Es handelt sich um ein epidemiologisches Pilotprojekt. Über einen Zeitraum von 3 Monaten (Januar bis März 2022) wurde in den Bereichen Station, Ambulanz, Konsildienst sowie bei den durchgeführten Operationen einer deutschen Universitätsklinik erhoben, wie viele Patientinnen und Patienten relativ im Verhältnis zur Gesamtanzahl der dort betreuten Patientinnen und Patienten eine Antibiotikagabe zur Therapie bzw. Prävention einer bakteriellen Infektion erhalten haben. Die Datenextraktion erfolgte immer um 15:00 Uhr nach 24 h und wurde von zwei unabhängigen Autoren geprüft. Nachfolgend erfolgte die Eingabe in das Statistikprogramm SPSS 26.0 (SPSS Inc., Armonk, NY, USA) und die Analyse mittels deskriptiver Darstellung der relativen Anzahl in Prozent.

## Ergebnisse

### Antibiotikaeinsatz in einzelnen Bereichen im Tagesverlauf über den Monat

**Figure 1:**
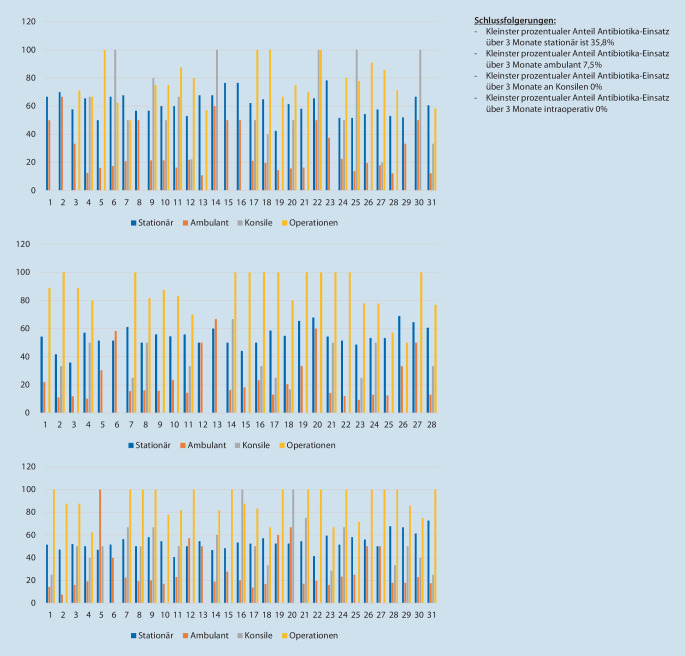

Zunächst erfolgte die monatliche Auswertung des Antibiotikaeinsatzes für bakterielle Infektionen in den Bereichen Station, Ambulanz, Konsildienst sowie Operationen. Die Ergebnisse sind in Abb. 1 graphisch dargestellt. Zusammenfassend ist der Antibiotikaeinsatz im stationären Bereich am höchsten. Der niedrigste prozentuale Anteil über die betrachteten drei Monate im stationären Bereich lag bei 35,8 %.

### Mittlerer Antibiotikaeinsatz im Tagesverlauf über den Monat

**Figure 2:**
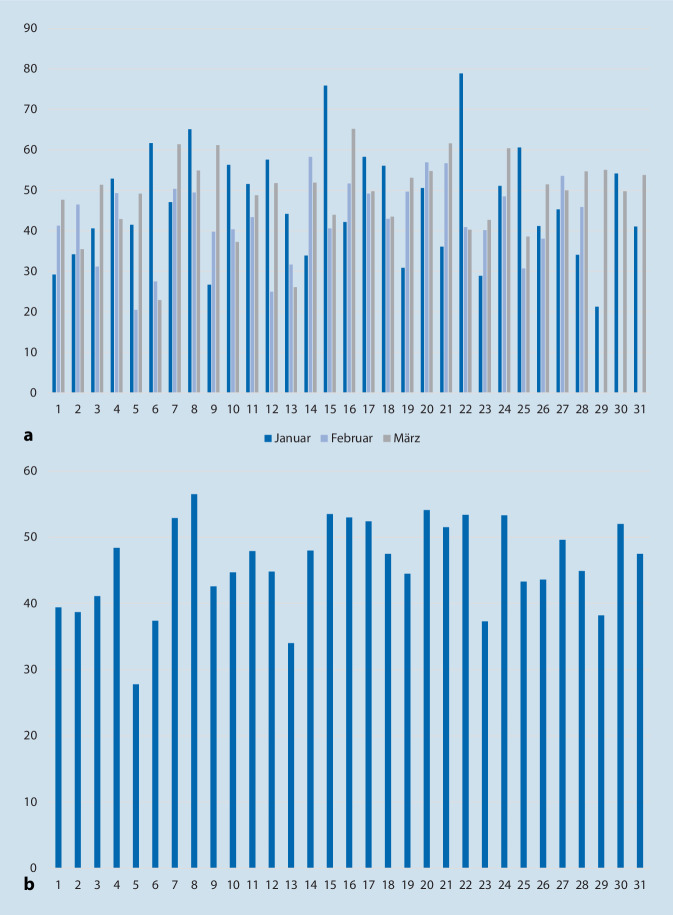

In einem zweiten Schritt wurden die Mittelwerte aus den drei betrachteten Monaten pro Tag gebildet sowie daraus die kumulativen Mittelwerte des Antibiotikaeinsatzes gegen bakterielle Infektionen. Kumulativ gesehen lag der höchste prozentuale Anteil bei 56,5 %, während der kleinste prozentuale Anteil 27,8 % betrug. Die Ergebnisse dieser Betrachtungen werden in Abb. 2 veranschaulicht.

### Kumulativer mittlerer Antibiotikaeinsatz pro Monat

**Figure 3:**
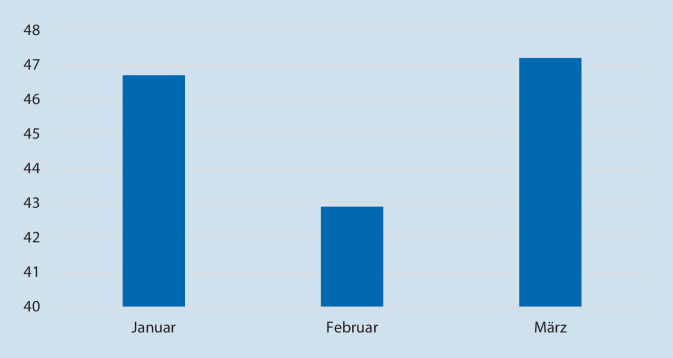

Schlussendlich wurde der gesamte mittlere kumulative Antibiotikaeinsatz pro Monat summiert. Dies ist in Abb. 3 dargestellt. Der geringste Anteil betrug dabei 42,9 % und der höchste 47,2 %. Tab. 1 gibt einen Überblick über die einzelnen kumulativen mittleren Antibiotikaverbrauch mit dem höchsten Einsatz auf Station sowie als Prophylaxe im Operationssaal.

**Table 1:** 

	Januar	Februar	März
Gesamt	46,7	42,9	47,2
Station	61,3	54,5	53,7
Ambulanz	28,2	24,6	29,2
Konsildienst	34,8	17,6	32,0
Operationssaal	54,9	75,0	77,9

Zusammenfassend erhalten etwa 40 % der urologischen Patienten dieser Universitätsklinik eine antibiotische Therapie bei bakterieller Harnwegsinfektion bzw. als perioperative Antibiotikaprophylaxe zur Vermeidung einer bakteriellen Infektion im Operationssaal.

## Diskussion

Wir haben in einem epidemiologischen Pilotprojekt untersucht, welchen relativen Anteil die antibiotische Therapie bzw. Prophylaxe im Verhältnis zur Gesamtpatientenzahl an einer deutschen Universitätsklinik hat. Zusammenfassend erhalten etwa 40 % der Patienten eine antibiotische Medikation, den größten Anteil machen dabei stationäre Patienten aus. Zwar erscheinen diese wesentlichen Ergebnisse nicht überraschend oder sind selbsterklärend, aber Lebentrau et al. schlussfolgerten in ihrer MR2-Umfrage, dass das Wissen von Urologen über infektiöse Erkrankungen und antibiotische Verschreibungspraxis unzureichend ist [11]. Zusätzlich konnten Wattengel et al. in ihrer retrospektiven Studie darstellen, dass 68 % der Antibiotika in der urologischen Ambulanz inadäquat eingesetzt werden [12, 13]. Obwohl die Urologie eben zu den Fachdisziplinen mit dem höchsten Antibiotikaverordnungsvolumen pro Tagesdosis gehört [11, 14]. Daher gibt es eine substantielle Notwendigkeit für eine gute Ausbildung über multiresistente Erreger und antibiotische Verschreibungspraxis. Dieses Wissen ist essentiell, um ABS-Programme überhaupt implementieren zu können [11].

Aus diesen Gründen wurde auch dieses Pilotprojekt initiiert, um die Notwendigkeit für die Beschäftigung mit Infektiologie in der Urologie nochmals heraus zu arbeiten, da sie einen signifikanten Anteil des Fachgebiets einnimmt. Kollegen sollen dafür sensibilisiert werden und soll identifiziert werden, wo der größte Anteil des Antibiotikaverbrauchs in der Urologie liegt, da dies einer der ersten Schritte zur Implementierung von ABS-Programmen ist.

Die ABS-Programme sind dabei in der Urologie wichtiger als je zuvor, da sie die Verbreitung von Infektionen mit multi-resistenten Erregern reduzieren können [13, 15–18]. In ihrem Rapid Review identifizierten Schneidewind et al. Schlüsselpunkte zur steigenden Bedeutung von ABS-Programmen bei Harnwegsinfektionen. Diese sind:

- 1. Trotz der bekannten Evidenz für ABS-Interventionen gibt es immer noch einen hohen Anteil von inadäquatem Antibiotikaeinsatz bei Harnwegsinfektionen und in der Urologie, insbesondere bei der asymptomatischen Bakteriurie und übermäßiger Verbrauch von Fluorchinolonen.
- 2. ABS-Programme haben das Potenzial, Therapieergebnisse zu verbessern und sowohl multiresistente Erreger als auch Krankenhauskosten zu senken.
- 3. Wichtige Angriffspunkte für ABS-Interventionen sind Einbeziehung eines Apothekers, strikte Leitlinienadhärenz, Verbesserung der bestehenden Leitlinien selbst, Verstehen der antibiotischen Verschreibungspraxis, insbesondere in der Notaufnahme, und Analyse der eigenen Surveillance Daten [13, 19].

Betont werden muss in diesem Kontext noch einmal die Leitlinienadhärenz, so schlussfolgerten Köves et al. in ihrer Analyse der GPIU(Global Prevalence Study of Infections in Urology)-Daten hinsichtlich der antibiotischen Prophylaxe bei Prostatastanzbiopsien: Eine stärkere Leitlinienadhärenz ist unbedingt notwendig, um die Patientenversorgung hinsichtlich antibiotischer Prophylaxen sowie Therapien zu verbessern [19, 20]. Weitere Argumente für ABS-Programme sind, dass Urologen selbst durch die Programme mehr über Infektionsmanagement lernen können, was wiederum zur Reduktion von inadäquatem Antibiotikaeinsatz führt und dabei steigen trotz dieser Reduktion infektiöse Komplikationen nicht an [21, 22].

Interessanterweise müssen die ABS-Interventionen und Ausbildung kontinuierlich aufrechterhalten werden, so konnten Jang et al. zeigen, dass nach Beendigung eines ABS-Programms die antibiotische Verschreibungspraxis sehr schnell auf das Ausgangsniveau zurück geht [23]. Laut Wathne et al. sind die fünf wesentlichen Angriffspunkte für ABS-Interventionen dabei: 1. Leitlinienadhärenz bei Therapieeinleitung, 2. Antibiotikaverschreibungspraxis in der Notaufnahme, 3. Nachvollziehen der Antibiotikatherapie von Patienten, die aus anderen Einrichtungen zugewiesen wurden, 4. Verstehen von kulturellen und institutionellen Gegebenheiten, die zur Antibiotikaverschreibung führen und 5. Dauer der Therapie in Tagen [24].

Ein weiterer interessanter Aspekt, der wenigen Urologen bekannt sein dürfte, ist, dass das Robert Koch-Institut bereits ein Programm zur Antibiotikaverbrauchssurveillance (ASV) durchführt. Diese Daten wären für Urologen und deren ABS-Interventionen besonders relevant, allerdings können daraus von außen keine Daten extrahiert werden [25]. Hier wären insbesondere Daten aus dem ambulanten Bereich ebenfalls interessant, da diese sich möglicherweise deutlich von dieser Erhebung an einer Universitätsklinik unterscheiden.

Außerdem ist zu diskutieren, dass es in den vier untersuchten Untergruppen, also Station, Ambulanz, Konsildienst sowie Operationssaal, erhebliche Unterschiede gibt. So handelt es sich im Operationssaal in der Regel um einen prophylaktischen Antibiotikaeinsatz, daher bleibt unsere Schlussfolgerung, dass der Antibiotikaeinsatz auf Station am höchsten ist, bestehen. Dies sollte in Folgestudien ebenfalls adressiert werden, insbesondere die Antibiotikaprophylaxe sollte getrennt von der Therapie eruiert werden.

Zusammenfassend sind ABS-Programme in der Urologie essentiell, da infektiologische Fragestellungen laut unseren Untersuchungen einen signifikanten Anteil unseres Fachgebiets ausmachen. Einschränkend ist zu bemerken, dass dieses Pilotprojekt lediglich bakterielle Entzündungen betrachtet hat, dabei ist die urologische Infektiologie wesentlich größer und beinhaltet z. B. auch virale Infektionen, Geschlechtskrankheiten oder Impfmedizin [26]. Weitere Limitationen dieser Arbeit liegen im Pilotcharakter dieser Untersuchung, so z. B. dass nur ein Universitätsklinikum betrachtet wurde, sodass von einem gewissen Selektionsbias auszugehen ist.

Aus diesem Pilotprojekt können aber Folgestudienentwickelt werden, die das Wissen über antibiotische Therapie in der Urologie verbessern. Dieses Wissen ist essentiell zur Implementierung von ABS-Programmen. Diese Folgestudien sollten sich auf die Verschreibungspraxis auf Station, da laut unseren Betrachtungen hier die meisten Antibiotika verbraucht worden sind, und eine genaue Analyse in den Notaufnahmen, da laut Wathne et al. hier 83,6 % der antibiotischen Therapien eingeleitet werden, fokussieren [24]. Weiterhin sollten Therapie und Prophylaxe getrennt betrachtet untersucht werden.

## Fazit für die Praxis

- Infektiologie macht einen signifikanten Anteil des Fachgebiets Urologie aus.
- Bakterielle Infektionen sind aufgrund der Häufigkeit und der Antibiotikaresistenzentwicklung von besonderer Bedeutung.
- Auf Station ist der Antibiotikaverbrauch am größten.
- Wissen über Antibiotikaeinsatz ist essentiell für die Implementierung von ABS(Antibiotic Stewardship)-Programmen.
- ABS-Interventionen können der Resistenzentwicklung nachweislich entgegenwirken.
- Folgeuntersuchungen sollten sich Antibiotikaverschreibungspraxis auf Station und in der Notaufnahme adressieren, denn dies sind bedeutungsvolle Angriffspunkte für die ABS-Intervention.
